# Deep Learning-Based Prediction of Diabetic Retinopathy Using CLAHE and ESRGAN for Enhancement

**DOI:** 10.3390/healthcare11060863

**Published:** 2023-03-15

**Authors:** Ghadah Alwakid, Walaa Gouda, Mamoona Humayun

**Affiliations:** 1 Department of Computer Science, College of Computer and Information Sciences, Jouf University, Sakakah 72341, Al Jouf, Saudi Arabia; gnalwakid@ju.edu.sa; 2 Department of Electrical Engineering, Faculty of Engineering at Shoubra, Benha University, Cairo 11672, Egypt; walaa.gouda@feng.bu.edu.eg; 3 Department of Information Systems, College of Computer and Information Sciences, Jouf University, Sakakah 72341, Al Jouf, Saudi Arabia

**Keywords:** vision loss, diabetic retinopathy, image enhancement, APTOS

## Abstract

Vision loss can be avoided if diabetic retinopathy (DR) is diagnosed and treated promptly. The main five DR stages are none, moderate, mild, proliferate, and severe. In this study, a deep learning (DL) model is presented that diagnoses all five stages of DR with more accuracy than previous methods. The suggested method presents two scenarios: case 1 with image enhancement using a contrast limited adaptive histogram equalization (CLAHE) filtering algorithm in conjunction with an enhanced super-resolution generative adversarial network (ESRGAN), and case 2 without image enhancement. Augmentation techniques were then performed to generate a balanced dataset utilizing the same parameters for both cases. Using Inception-V3 applied to the Asia Pacific Tele-Ophthalmology Society (APTOS) datasets, the developed model achieved an accuracy of 98.7% for case 1 and 80.87% for case 2, which is greater than existing methods for detecting the five stages of DR. It was demonstrated that using CLAHE and ESRGAN improves a model’s performance and learning ability.

## 1. Introduction

The progressive eye disease known as DR is a direct result of having mellitus. Increases in blood glucose occur chronically in people with diabetes mellitus where the pancreas does not generate or release enough blood adrenaline [1,2]. Most diabetics go blind from DR, especially those of retirement age in low-income nations. Early identification is crucial for preventing the consequences that can arise from chronic diseases such as diabetes [3,4].

**Figure 1:**
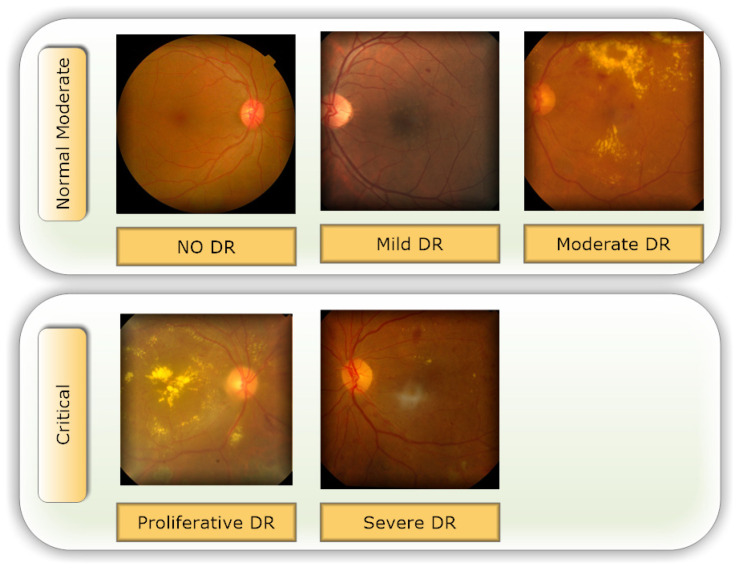
The five phases of diabetic retinopathy, listed by severity.

Retinal vasculature abnormalities are the hallmark of DR, which can progress to irreversible vision loss due to scarring or hemorrhage [1,5]. This may cause gradual vision impairment and, in its most severe form, blindness. It is not possible to cure the illness, so treatment focuses on preserving the patient’s present level of eyesight [6,7]. In most cases, a patient’s sight may be saved if DR is diagnosed and treated as soon as possible. In order to diagnose DR, an ophthalmologist should inspect images of the retina manually, which is an expensive and time-consuming process [8]. The majority of ophthalmologists today still use the tried-and-true method of analyzing retinal pictures for the presence and type of different abnormalities in order to diagnose DR. Microaneurysms (MIA), hemorrhages (HEM), soft exudates (SOX), and hard exudates (HEX) are the four most common forms of lesions identified [1,9], which can be identified as the following:

- In earlier DR, MA appear as tiny, red dots on the retina due to a weakening in the vessel walls. The dots have distinct borders and a dimension of 125 μm or less. There are six subtypes of microaneurysms, but the treatment is the same for all of them [10,11].
- In contrast to MA, HM are characterized by big spots on the retina with uneven edge widths of more than 125 μm. A hemorrhage can be either flame or blot, according to whether the spots are on the surface or deeper in the tissue [12,13].
- The swelling of nerve fibers causes soft exudates, which appear as white ovals on the retina as defined as SX [1,9].
- Yellow spots on the retina, known as EX, are the result of plasma leakage. They extend across the periphery of the retina and have defined borders [1,2].
- Lesions caused by MA and HM tend to be red, while blemishes caused by the two forms of exudates tend to be bright. There are five distinct stages of DR that can be detected: no DR, mild DR, moderate DR, severe DR, and proliferative DR [13], as shown in Figure 1.
- For DR diagnosis to be performed manually, experts in the field are needed, even though the most expert ophthalmologists have problems due to DR variability. Accurate machine learning techniques for automated DR detection have the ability to those defects [2,8].
- Our objective was to develop a quick, fully automated DL based DR categorization that may be used in practice to aid ophthalmologists in assessing DR. DR can be prevented if it is detected and treated quickly after it first appears. To achieve this goal, we trained a model using innovative image preprocessing techniques and an Inception-V3 [14,15] model for diagnosis using the publicly available APTOS dataset [16].

Below, we highlight the original contributions of our study.

- To generate high-quality images for the APTOS dataset, we used the (CLAHE) [17] filtering algorithm in conjunction with enhanced super-resolution generative adversarial networks (ESRGAN) [18], which is the main contribution of the presented work.
- By employing the technique of augmentation, we ensured that the APTOS dataset contained a consistent amount of data.
- Accuracy (*Acc*), confusion matrix (CM), precision (*Prec*), recall (*Re*), top n accuracy, and the *F*1-score (*F1sc*) were the indicators used in a comprehensive comparative study to determine the viability of the proposed system.
- Pre-trained networks trained on the APTOS data set were fine-tuned with the use of an Inception-V3 weight-tuning algorithm.
- By adopting a varied training procedure backed by various permutations of training strategies, the general reliability of the suggested method was enhanced, and overfitting was avoided (e.g., learning rate, data augmentation, batch size, and validation patience).
- The APTOS dataset was used during both the training and evaluation phases of the model’s development. By employing stringent 80:20 hold-out validation, the model achieved a remarkable 98.71% accuracy of classification using enhancement techniques and 80.87% without using enhancement techniques.

This research presents two cases scenarios. In case 1, an optimal technique for DR stage enhancement using CLAHE followed by ESRGAN techniques was developed. In case 2 no enhancement was applied to the images. Due to the class imbalance in the dataset, oversampling was required using augmentation techniques. In addition, we trained the weights of each model using Inception-V3, and the results of the models were compared using APTOS dataset images. Section 2 provides context for the subsequent discussion of the related work. Section 4 presents and analyzes the results of the technique described in Section 3, and Section 5 summarizes the research.

## 2. Related Work

There are various issues with DR picture detection when done manually. Numerous patients in underdeveloped nations face challenges due to a shortage of competence (trained ophthalmologists) and expensive tests. Because of the importance of timely detection in the fight against blindness, automated processing methods have been devised to facilitate accessibility for accurate and speedy diagnosis and treatment. Automated DR classification accuracy has recently been achieved by Machine Learning (ML) models trained on ocular fundus pictures. A lot of work has gone into developing automatic methods that are both efficient and inexpensive [19,20,21].

This means that these methods are now universally superior to their traditional counterparts. Following, we present a deeper examination of the two primary schools of thought in DR categorization research: classical, specialist approaches, and state-of-the-art, machine-learning-based approaches. For instance, Kazakh-British et al. [22], performed experimental studies with a relevant processing pipeline that extracted arteries from fundus pictures, and then a CNN model was trained to recognize lesions. Other work presented by Alexandr et al. [23] contrasted two widely-used classic designs (DenseNet and ResNet) with a new, enhanced structure (EfficientNet). Use of the APTOS symposium dataset allowed for the retinal image to be classified into one of five categories. Local binary convolutional neural network (LBCNN) deterministic filter generation was introduced by Macsik et al. [24] which mimicked the successfulness of the CNN with a smaller training set and less memory utilization, making it suitable for systems with limited memory or computing resources. Regarding binary classification of retinal fundus datasets into healthy and diseased groups, they compared their method with traditional CNN and LBCNN that use probabilistic filter sequence.

Al-Antary & Yasmine [19] suggested a multi-scale attention network (MSA-Net) for DR categorization. The encoder network embeds the retina image in a high-level representational space, enriching it with mid- and high-level characteristics. A multi-scale feature pyramid describes the retinal structure in another location. In addition to high-level representation, a multi-scale attention mechanism improves feature representation discrimination. The model classifies DR severity using cross-entropy loss. The model detects healthy and unhealthy retina pictures as an extracurricular assignment using weakly annotations. This surrogate task helps the model recognize non-healthy retina pictures. EyePACS and APTOS datasets performed well with the proposed technique. Medical DR identification was the focus of an investigation by Khalifa et al. [25] on deep transfer learning models. A series of experiments was conducted with the help of the APTOS 2019 dataset. Five different neural network architectures (AlexNet, Res-Net18, SqueezeNet, GoogleNet, VGG16, and VGG19) were used in this research. Selecting models with fewer layers than DenseNet and Inception-Resnet was a key factor. Model stability and overfitting were both enhanced by additional data. Hemanth et al. [26] presented a convolutional neural network–based approach to DR detection and classification. They employed HIST and CLAHE to improve contrast in the images, and the resulting CNN model achieved 97% accuracy in classification and a 94% F-measure. Maqsood et al. [27] introduced a new 3D CNN model to localize hemorrhages, an early indicator of DR, using a pre-trained VGG-19 model to extract characteristics from segmented hemorrhages. Their studies used 1509 photos from HRF, DRIVE, STARE, MESSIDOR, DIARETDB0, and DIARETDB1 databases and averaged 97.71% accuracy. Das et al. [28] suggested a unique CNN for categorizing normal and abnormal patients utilizing the fundus images. The blood arteries were recovered from the images using a maximal principal curvature approach. Adaptive histogram equalization and morphological opening were used to correct improperly segmented regions. The DIARETDB1 dataset was considered, and an accuracy and precision of 98.7% and 97.2%, respectively, was attained.

Wang et al. [29] created Lesion-Net to improve the encoder’s representational power by including lesion detection into severity grading. InceptionV3 trained and verified the design. Liu et al. [30] used TL with different models to investigate DR from EyePACS. A new cross-entropy loss function and three hybrid model structures classified DR with 86.34% accuracy.

Table 1 summarizes the many attempts to detect DR anomalies in photos using various DL techniques [19,24,31,32,33,34,35,36,37]. According to the results of the research into DR identification and diagnostic methods, there are still a lot of loopholes that need to be investigated. For example, there has been minimal emphasis on constructing and training a bespoke DL model entirely from the beginning because of a lack of a large amount of data, even though numerous researchers have obtained excellent dependability values with pre-trained models using transfer-learning.

Ultimately, training DL models with raw images instead of preprocessed images severely restricts the final classification network’s scalability, as was the case in nearly all of these studies. In order to resolve these problems, the current research created a lightweight DR detection system by integrating multiple layers into the architecture of pre-trained models. This leads to a more efficient and effective proposed system that meets users’ expectations.

## 3. Research Methodology

**Figure 2:**
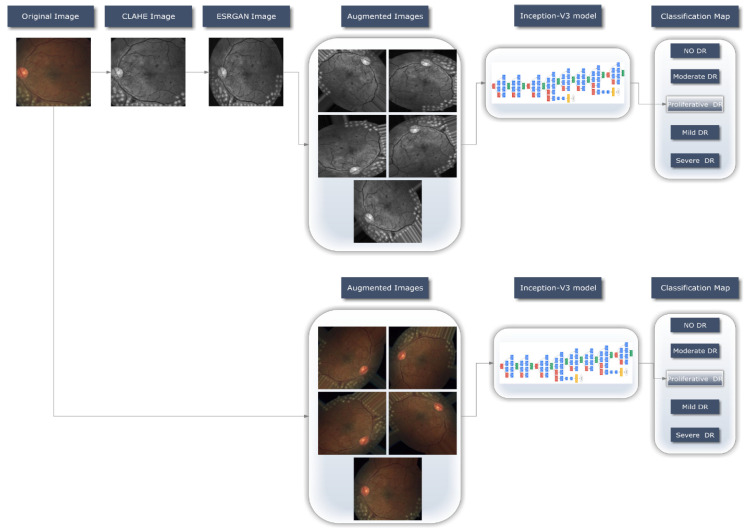
An illustration of the DR detecting system process.

For the DR detection system to operate, as shown in Figure 2, a transfer DL strategy (Inception-V3) was retrained in the image dataset to learn discriminative and usable feature representations. This section offers a concise summary of the method followed when working with the provided dataset. The preprocessing stage is then clearly outlined, and implementation specifics of the proposed system are covered. These include the two cases scenarios used in this context, the preprocessing techniques proposed, the basic design, and the training methodology for the approach that was ultimately chosen.

### 3.1. Data Set Description

Selecting a dataset with a sufficient number of high-quality photos is crucial. This study made use of the APTOS 2019 (Asia Pacific Tele-Ophthalmology Society) Blindness Detection Dataset [16], a publicly available Kaggle dataset that incorporates a huge number of photos. In this collection, high-resolution retinal pictures are provided for the five stages of DR, classified from 0 (none) to 4 (proliferate DR), with labels 1–4 corresponding to the four levels of severity. There are 3662 retinal pictures in total; 1805 are from the “no DR” group, 370 are from the “mild DR” group, 999 are from the “moderate DR” group, 193 are from the “severe DR” group, and 295 are from the “proliferate DR” group, as illustrated in Table 2. Images are 3216 × 2136 pixels in size, and Figure 1 shows some examples of these kind of pictures. There is background noise in the photographs and the labels, much like any real-world data set. It is possible that the provided images will be flawed in some way, be it with artifacts, blurriness, improper exposure, or some other issue. The photos were collected over a long period of time from a number of different clinics using different cameras, all of which contribute to the overall high degree of diversity.

### 3.2. Proposed Methodology

An automatic DR classification model was developed using the dataset referenced in this paper; its general process is demonstrated in Figure 1. It demonstrates two different scenarios: case 1 in which the preprocessing step is performed using CLAHE followed by ESRGAN is used, and case 2 in which neither step is performed, while using augmentation of the images to prevent overfitting in both scenarios. Lastly, images were sent into the Inception-V3 model for classification step.

#### 3.2.1. Preprocessing Using CLAHE and ESRGAN

Images of the retinal fundus are often taken from several facilities using various technologies. Consequently, given the high intensity variation in the photographs used by the proposed method, it was crucial to enhance the quality of DR images and get rid of various types of noise. All images in case 1 underwent a preliminary preprocessing phase prior to augmentation, and training necessitated various stages:

1. CLAHE
2. Resize each picture to 224 × 224 × 3 pixels.
3. ESRGAN
4. Normalization

**Figure 3:**
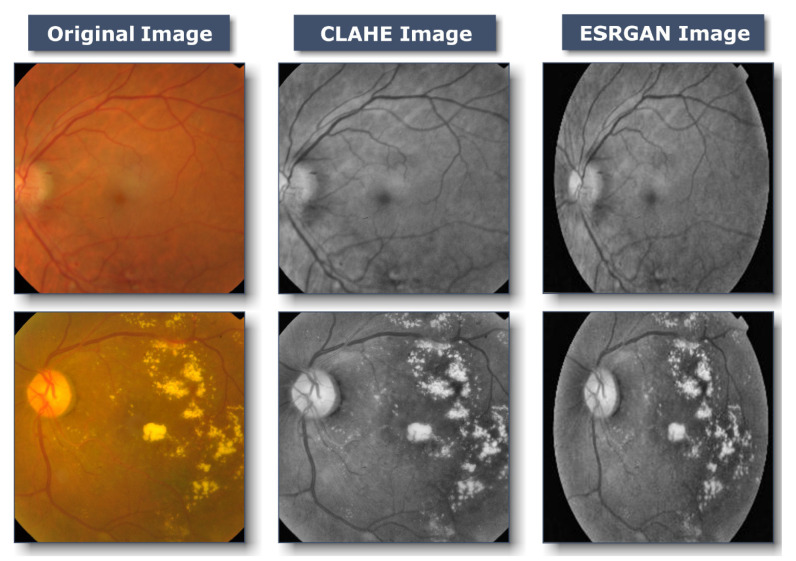
Samples of the proposed image-enhancement techniques: original, unedited image; then rendition of this same image with CLAHE; finally final enhanced image after applying ESRGAN.

**Figure 4:**
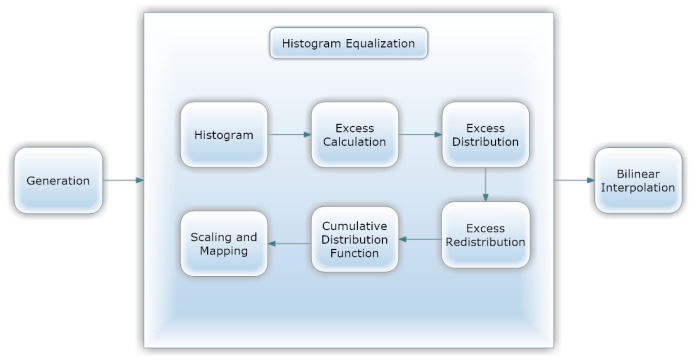
CLAHE architecture.

**Figure 5:**
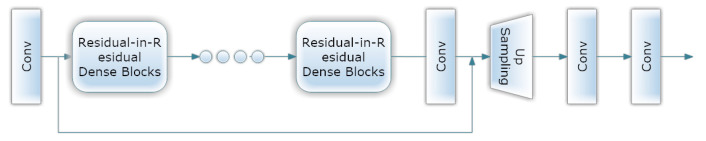
ESRGAN architecture.

Figure 3 shows that first, CLAHE (shown in Figure 4) was used to improve the DR image’s fine details, textures, and low contrast by redistributing the input image’s lightness values [38]. Utilizing CLAHE, the input image was first sectioned into four small tiles. Each tile underwent histogram equalization with a clip limit, which involved five steps: computation, excess calculation, distribution, redistribution, and scaling and mapping using a cumulative distribution function (CDF). For each tile, a histogram was calculated, where bins value above the clip limit were aggregated and spread to other bins. Histogram values were then calculated using CDF for the input image pixel scale and then mapped tile to CDF values. To boost contrast, bilinear interpolation stitched the tiles together [39]. This technique improved local contrast enhancement while also making borders and slopes more apparent. Following this, all photos were scaled to suit the input of the learning model, which was 224 × 224 × 3. Figure 3 depicts the subsequent application of ESRGAN on the output of the preceding stage. ESRGAN [40] (shown in Figure 5) pictures can more closely mimic image artifacts’ sharp edges [41]. To improve performance, ESRGAN adopted the basic architecture of SRResNet, in which Residual-in-Residual Dense Blocks are substituted for the traditional ESRGAN basic blocks, as shown in Figure 5. Intensity differences between images can be rather large, thus images were normalized so that their intensities fell within the range −1 to 1. This kept the data within acceptable bounds and removed noise. As a result of normalization, the model was less sensitive to variations in weights, making it easier to tune. Since the method shown in Figure 3 improved the image’s contrast while simultaneously emphasizing the image’s boundaries and arcs, it yielded more accurate findings.

#### 3.2.2. Data Augmentation

Data augmentation was implemented on the training set to increase the number of images and alleviate the issue of an imbalanced dataset before exposing Inception-V3 to the dataset images. In most cases, deeper learning models perform better when given more data to learn from. We can utilize the characteristics of DR photos by applying several modifications to each image. A deep neural network (DNN) is unaffected by any changes made to the input image, including scaling it up or down, flipping it horizontally or vertically, or rotating it by a certain number of degrees. Regulating the data, minimizing overfitting, and rectifying imbalances in the dataset are all accomplished through the use of data augmentations (i.e., shifting, rotating, and zooming). One of the transformations used in this investigation was horizontal shift augmentation, which involves shifting the pixels of an image horizontally while maintaining the image’s aspect ratio, with the step size being specified by an integer between 0 and 1. Another kind of transformation is rotation, in which the image is arbitrarily rotated by an angle between 0 and 180 degrees. To create fresh samples for the network, all prior alterations to the training set’s images were applied.

**Figure 6:**
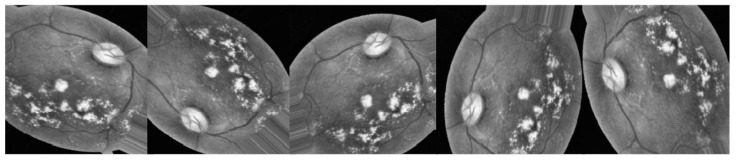
Illustrations of the same image, augmented with enhancement.

**Figure 7:**
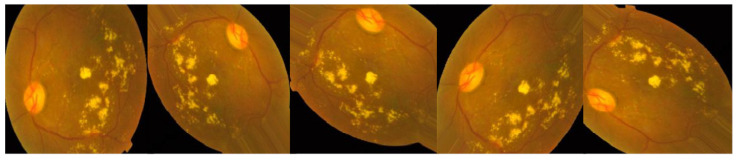
Illustrations of the same image augmented without enhancement.

In this study, two scenarios were utilized to train Inception-V3. The first was to apply augmentation to the enhanced images, as depicted in Figure 6, and the second was to apply augmentation to the raw images, as depicted in Figure 7. Both Figure 4 and Figure 5 are attempts to expand data volume by making slightly modified copies of current data or by synthesizing data generated from existing data while keeping all other parameters constant (Figure 4 and Figure 5), with the same total number of images in both cases.

**Figure 8:**
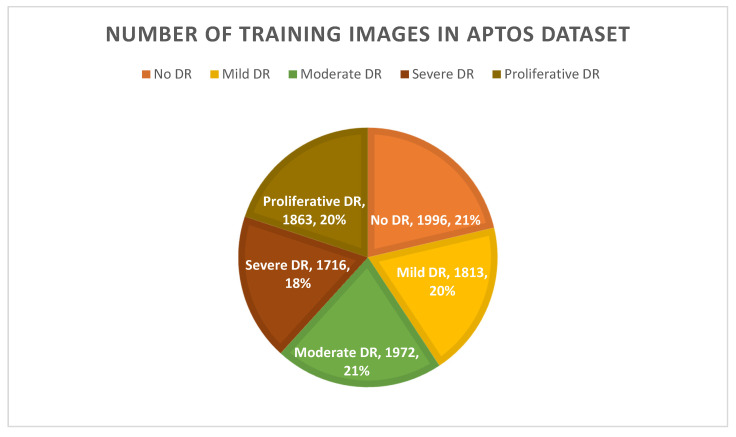
Number of training images after using augmentation techniques.

In a second use of data augmentation techniques, the issues of inconsistent sample sizes and complicated classifications were resolved. As seen in Table 2, the APTOS dataset exemplifies the “imbalanced class” because the samples are not distributed evenly throughout the several classes. After applying augmentation techniques to the dataset, the classes are obviously balanced for both scenarios, as depicted in Figure 8.

#### 3.2.3. Learning Model (Inception-V3)

In this section, the approach’s fundamental theory is outlined and explained. Inception-v3 [11,12] is among transfer learning pretrained models, superseding the original architecture for Inception-v1 [42] and Inception-v2 [43]. The Inception-v3 model is trained using the ImageNet datasets [44,45], which contain the information required for identifying one thousand classes. The error rate for the top five in ImageNet is 3.5%, while the error rate for the top one was lowered to 17.3%.

Inception was influenced in particular by technique of Serre et al. [46], which processes information in several stages. By adopting the Lin et al. [47] method, the developers of Inception were able to improve the model precision of the neural networks, making them a significant design requirement. As a result of the dimension reduction to 1*1 convolutions, this also protected them from computing constraints. Researchers were able to significantly reduce the amount of time and effort spent on DL picture classification using Inception [48]. Using only the theoretical explanations offered by Arora et al. [49], they emphasized discovering an optimal spot between the typical technique of improving performance—increasing both depth and size—and layer separability. When utilized independently, both procedures are computationally expensive. This was the fundamental goal of the 22-layer architecture employed by the Inception DL system, in which all filters are learned. On the basis of research by Arora et al. [49], a correlation statistical analysis was developed to generate highly associated categories that were input into the subsequent layer. The 1 × 1 layer, the 3 × 3 layer, and the 5 × 5 convolution layer were all inspired by the concept of multiscale processing of visual data. Each of these layers eventually becomes a set of 1 × 1 convolutions [48] following a process of dimension reduction.

## 4. Experimental Results

### 4.1. Instruction and Setup of Inception-V3

To demonstrate the effectiveness of the deployed DL system and to compare results to industry standards, tests were carried out on the APTOS dataset. The dataset was divided into three categories in accordance with the suggested training method. Eighty percent of the data was utilized for training (9952 photographs), ten percent for testing (1012 photos), and the remaining ten percent was randomly selected and used as a validation set (1025 photos) to evaluate performance and save the best weight combinations. All photographs were reduced in size during the training process to 224 × 224 × 3 pixel resolution. We tested the proposed system’s TensorFlow Keras implementation on a Linux desktop equipped with a GPU RTX3060 and 8 GB of RAM.

Using the Adam optimizer and a method that slows down training when learning has stalled for too long, the proposed framework was first trained on the APTOS dataset (i.e., validation patience). Throughout the entirety of the training process, hyperparameters were input into the Adam optimizer. We used a range of 1 × 10^3^ to 1 × 10^5^ for the learning rate, 2–64 for the batch size (with an increase of 2× the previous value), 50 epochs, 10 for patience, and 0.90 for momentum. Our arsenal of anti-infectious measures was completed by a method known as “batching” for the dissemination of infectious forms.

### 4.2. Evaluative Parameters

This section describes the evaluation methods and their results. Classifier accuracy (*Acc*) is a standard performance measure. It is determined by dividing the number of successfully categorized instances (images) by the total number of examples in the dataset (Equation (1)). Picture categorization systems are often evaluated using precision (*Prec*) and recall (*Re*). As demonstrated in Equation (2), precision improves with the number of accurately labeled photos, whereas recall is the ratio of properly categorized images in the dataset to those related numerically (3). The higher the *F*1-score, the more reliable the system is at making predictions about the future. The *F*1-score can be determined using Equation (4), (*F1sc*). With respect to the study’s last criterion, top N accuracy, it was found that the highest probability answers from model N should coincide with the expected softmax distribution. An accurate classification is made if at least one of N predictions corresponds to the target label.

(1) $$Accuracy=\frac{T^{p}+T^{n}}{T^{p}+T^{n}+F^{p\;}+F^{n}}$$

(2) $$Precision=\frac{T^{p}}{T^{p}+F^{p\;}}$$

(3) $$Recall=\frac{T^{p}}{T^{p}+F^{n\;}}$$

(4) $$F1\text{-}score=2*\left(\frac{Prec*Re}{Prec+Re}\right)$$

True positives, represented by the symbol (*T^p^*), are successfully anticipated positive cases, and true negatives (*T^n^*) are effectively predicted negative scenarios. False positives (*F^p^*) are falsely predicted positive situations, whereas false negatives (*F^n^*) are falsely projected negative situations.

### 4.3. Performance of Inception-V3 Model Outcomes

Considering the APTOS dataset, two distinct cases sets were investigated, in which Inception-V3 was applied to our dataset in two distinct scenarios, the first with enhancement (CLAHE + ESRGAN) and the second without enhancement (CLAHE + ESRGAN), as depicted in Figure 2. We split it up this way to cut down on the total amount of time needed to conduct the project. To train a model, 50 epochs were used, with learning rates ranging from 1 × 10^3^ to 1 × 10^5^, and batch sizes varying from 2 to 64. To achieve the highest possible level of precision, Inception-V3 was further tweaked by freezing between 140 and 160 layers. Several iterations of the same model with the same parameters were used to generate a model ensemble, since random weights were generated for each iteration, the precision fluctuated from iteration to iteration. Mean and standard deviation statistics for this procedure are displayed in Table 3 and Table 4, respectively, for the cases where the first 143 layers were frozen with CLAHE + ESRGAN and the cases where they were not.

**Figure 9:**
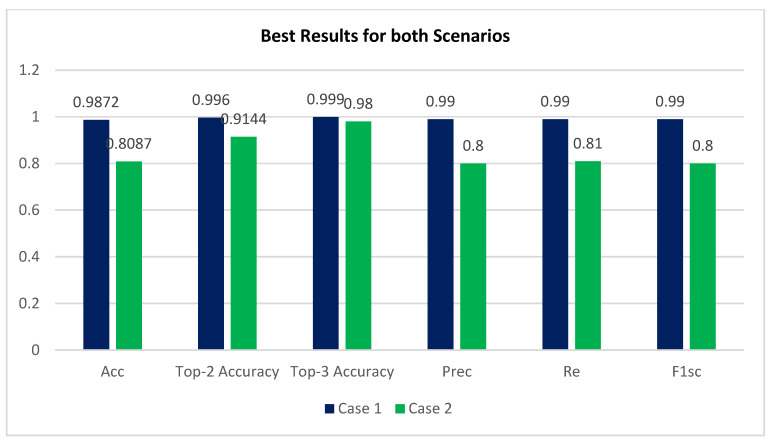
Best results for both scenarios.

The top performance from each iteration was saved and is shown in Table 5 and Table 6, for case 1 and case 2, respectively, revealing that the best results produced with and without preprocessing using CLAHE + ESRGAN were 98.7% and 80.87%, respectively. Figure 9 depicts the optimal outcome for the two scenarios based on the utilized evaluation metrics case 1 using CLAHE and ESRGAN, and case 2 without using them.

**Figure 10:**
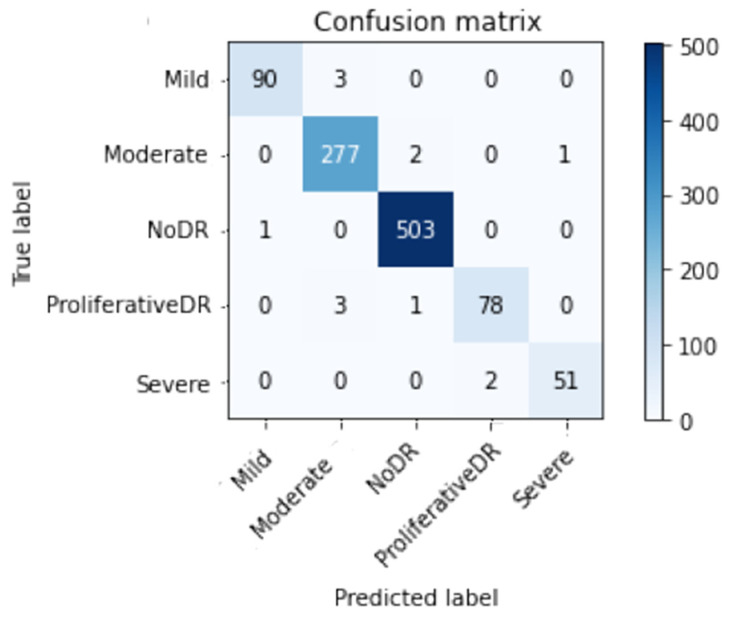
Best confusion matrix of Inception-V3 with enhancement (with CLAHE + ESRGAN).

**Figure 11:**
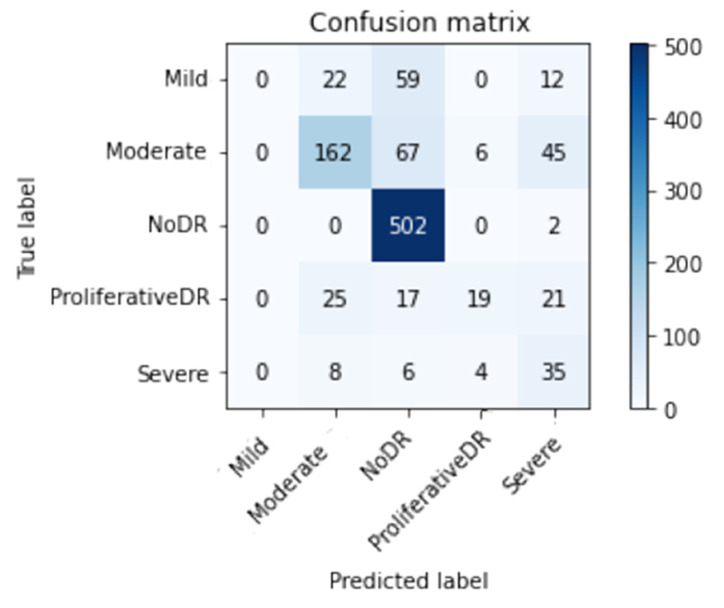
Best confusion matrix of Inception-V3 without enhancement (without CLAHE + ESRGAN).

Figure 10 and Figure 11 show the confusion matrix with and without using CLAHE + ESRGAN, respectively.

Table 7 and Table 8 show the total number of photos utilized for testing in each class for the APTOS dataset. According to the data, it is clear that the No DR class has the most images with 504, and its *Prec*, *Re*, and *F1sc* give the highest values of 99 100 and 100% for case 1, and 97, 97, and 97% for case 2.

Using retinal pictures to improve the accuracy with which ophthalmologists identify infections, while reducing their effort, was demonstrated to be practical in real-world scenarios.

### 4.4. Evaluation Considering a Variety of Other Methodologies

Effectiveness was compared to that of other methods. According to Table 9, our method exceeds other alternatives in terms of effectiveness and performance. The proposed inception model achieved an overall accuracy rate of 98.7%, surpassing the present methods.

## 5. Discussion

Based on CLAHE and ESRGAN, a novel DR categorization scheme is presented in this research. The developed model was tested on the DR images founded in the APTOS 2019 dataset. There were two training scenarios: case 1 with CLAHE + ESRGAN applied to the APTOS dataset, and case 2 without CLAHE + ESRGAN. Through 80:20 hold-out validation, the model attained a five-class accuracy rate of 98.7% for case 1 and 80.87% for case 2. The proposed method classified both cases scenarios using the pretrained Inception-V3 infrastructure. Throughout model construction, we evaluated the classification performance of two distinct scenarios and found that enhancement techniques produced the best results (Figure 9). The main contributing element in our methodology was the general resolution enhancement of CLAHE + ESRGAN, which we proved, with evidence, is responsible for the great improvement in the accuracy.

## 6. Conclusions

By identifying retinal images displayed in the APTOS dataset, we established a strategy for quickly and accurately diagnosing five distinct forms of cancer. The proposed method employs case 1 with images enhanced with CLAHE and ESRGAN, and case 2 with original images. The case 1 scenario employs four-stage picture enhancement techniques to increase the image’s luminance and eliminate noise. CLAHE and ESRGAN were the two stages with the best impact on accuracy, as demonstrated by experimental results. State-of-the-art techniques in preprocessed medical imagery were employed to train Inception-V3 with augmentation techniques that helped reduce overfitting and raised the entire competencies of the suggested methodology. This solution showed that when using Inception-V3, the conception model achieved a correctness of 98.7% ≈ 99% for the case 1 scenario and 80.87% ≈ 81% for the case 2 scenario, both of which are in line with the accuracy of trained ophthalmologists. The use of CLAHE and ESRGAN in the preprocessing step further contributed to the study’s novelty and significance. The proposed methodology outperformed established models, as evidenced by a comparison of their respective strengths and weaknesses. To prove the effectiveness of the proposed method, it must be tested on a sizable and intricate dataset, ideally consisting of a significant number of potential DR instances. In the future, new datasets may be analyzed using DenseNet, VGG, or ResNet, as well as additional augmentation approaches. Additionally, ESRGAN and CLAHE can be conducted independently to determine their impact on the classification procedure.

## Figures and Tables

**Table 1:** A review of the literature comparing several DR diagnostic techniques.

Reference	Year	Technique	Total Number of Images	Classes	Dataset	Accuracy	Precision	Recall	Receiver Operating Characteristic ROC
[19]	2021	Multi-scale attention network (MSA-Net)		5	APTOS	84.6%	90.5%	91%	-
					Eyepacs	87.5%	78.7%	90.6%	76.7%
[24]	2022	Local binary convolutional neural network (LBCNN)		2	APTOS	97.41%	96.59%	94.63%	98.71%
[31]	2022	Support vector machine (SVM)	Test: 1928	2	APTOS	94.5%		75.6%	
			Test: 103		IDRiD	93.3%		78.5%	
[32]	2022	CNN		2	APTOS	95.3%			
[33]	2022	Inception-ResNet-v2		5	APTOS	82.18%			
[34]	2021	Squeeze Excitation Densely Connected deep CNN		5	APTOS			96%	
					EyePACS			93%	
[35]	2021	VGG-16	Test = 1728	5	APTOS	74.58%			
[36]	2022	VGG16	13,626	2	APTOS	73.26%	99%	99%	
		DenseNet121				96.11%			
[37]	2022	DenseNet201	3662	5	APTOS	93.85%	90.90%	80.60%	
			2355	3	New Dataset	94.06%	94.74%	94.45%	

**Table 2:** Class-Wide Image Distribution.

Class Index	DR Level	# Images
0	No DR	1805
1	Mild DR	370
2	Moderate DR	999
3	Severe DR	193
4	Proliferate DR	295

# = number of images.

**Table 3:** Average and standard deviation accuracy with enhancement (CLAHE + ESRGAN).

Batch Size	Learning Rate	Accuracy	Mean	Standard Deviation
2	0.00001	0.983202	0.982543	0.001140989
	0.0001	0.983202		
	0.001	0.981225		
4	0.00001	0.982213	0.982213	0
	0.0001	0.982213		
	0.001	0.982213		
8	0.00001	0.982213	0.980237	0.008088282
	0.0001	0.987154		
	0.001	0.971344		
16	0.00001	0.980237	0.980896	0.001141024
	0.0001	0.982213		
	0.001	0.980237		
32	0.00001	0.979249	0.979249	0.000988126
	0.0001	0.978261		
	0.001	0.980237		
64	0.00001	0.978261	0.977931	0.000570495
	0.0001	0.978261		
	0.001	0.977273		

**Table 4:** Average and standard deviation accuracy without enhancement (CLAHE + ESRGAN).

Freeze	Batch Size	Learning Rate	Accuracy	Mean	Standard Deviation
140	2	0.00001	0.779599	0.761992	0.021731047
		0.0001	0.76867		
		0.001	0.737705		
	4	0.00001	0.783242	0.780814	0.005855271
		0.0001	0.785064		
		0.00001	0.774135		
	8	0.00001	0.777778	0.780814	0.002782382
		0.0001	0.781421		
		0.001	0.783242		
	16	0.00001	0.790528	0.7881	0.004206547
		0.0001	0.783242		
		0.001	0.790528		
	32	0.00001	0.786885	0.788707	0.01014166
		0.0001	0.799636		
		0.001	0.779599		
	64	0.00001	0.794171	0.798421	0.008985212
		0.0001	0.808743		
		0.001	0.79235		

**Table 5:** Best accuracy with enhancement (CLAHE + ESRGAN).

*Acc*	*Prec*	*Re*	*F1sc*	Top-2 Accuracy	Top-3 Accuracy
0.9872	0.99	0.99	0.99	0.996	0.999

**Table 6:** Best accuracy without enhancement (CLAHE + ESRGAN).

*Acc*	*Prec*	*Re*	*F1sc*	Top-2 Accuracy	Top-3 Accuracy
0.8087	0.80	0.81	0.80	0.9144	0.9800

**Table 7:** Detailed results for each class using CLAHE + ESRGAN.

	*Prec*	*Re*	*F1sc*	Total Images
Mild DR	0.99	0.97	0.98	93
Moderate DR	0.98	0.99	0.98	280
No DR	0.99	1.00	1.00	504
Proliferative DR	0.97	0.95	0.96	82
Severe DR	0.98	0.96	0.97	53
Average	0.99	0.99	0.99	1012

**Table 8:** Detailed results for each class without using CLAHE + ESRGAN.

	*Prec*	*Re*	*F1sc*	Total Images
Mild DR	0.58	0.62	0.60	93
Moderate DR	0.70	0.78	0.74	280
No DR	0.97	0.97	0.97	504
Proliferative DR	0.68	0.48	0.56	82
Severe DR	0.43	0.31	0.36	53
Average	0.80	0.81	0.80	1012

**Table 9:** Comparison of system performance to previous research using the APTOS Dataset.

Reference	Technique	Accuracy
[19]	MSA-Net	84.6%
[24]	*LBCNN*	97.41%
[31]	SVM	94.5%
[32]	CNN	95.3%
[33]	Inception-ResNet-v2	97.0%,
[35]	VGG-16	74.58%
[36]	VGG16	73.26%
	DenseNet121	96.11%
[37]	DenseNet201	93.85%
[50]	Vision Transformer, Bidirectional Encoder representation for image Transformer, Class-Attention in Image Transformers, Data efficient image Transformers	94.63%
[51]	EfficientNet-B6	86.03%
[52]	SVM classifier and MobileNet_V2 for feature extraction	88.80%
[53]	Densenet-121, Xception, Inception-v3, Resnet-50	85.28%
[54]	Inception-ResNet-v2	72.33%
[55]	MobileNet_V2	93.09%
[56]	EfficientNet and DenseNet	96.32%
[57]	VGG16	96.86%
[58]	Resnet-50	77.22%
[59]	Hybrid Residual U-Net	94%
[60]	Inception-v3	88.1%
Proposed Methodology	Inception-V3 (without using CLAHE + ESRGAN) Case 2	80.87%
	Inception-V3 (using CLAHE + ESRGAN) Case 1	98.7%

## Data Availability

Will be furnished on request.
